# Large Omnivore Movements in Response to Surface Mining and Mine Reclamation

**DOI:** 10.1038/srep19177

**Published:** 2016-01-11

**Authors:** Bogdan Cristescu, Gordon B. Stenhouse, Mark S. Boyce

**Affiliations:** 1 Department of Biological Sciences, University of Alberta Edmonton, Alberta, T6G 2E9, Canada; 2 Grizzly Bear Program, Foothills Research Institute Box 6330, Hinton, Alberta T7V 1X7, Canada; 3 Department of Biological Sciences, University of Alberta Edmonton, Alberta, T6G 2E9, Canada

## Abstract

Increasing global demands have resulted in widespread proliferation of resource extraction. Scientists are challenged to develop environmental mitigation strategies that meet societal expectations of resource supply, while achieving minimal disruption to sensitive “wilderness” species. We used GPS collar data from a 9-year study on grizzly bears (*Ursus arctos*) (*n* = 18) in Alberta, Canada to assess movements and associated space use during versus after mining. Grizzly bear home range overlap with mined areas was lower during active mining except for females with cubs, that also had shortest movements on active mines. However, both females with cubs and males made shorter steps when on/close to mines following mine closure and reclamation. Our results show differences in bear movement and space-use strategies, with individuals from a key population segment (females with cubs) appearing most adaptable to mining disturbance. Preserving patches of original habitat, reclaiming the landscape and minimizing the risk of direct human-induced mortality during and after development can help conserve bears and other wildlife on industrially modified landscapes.

For many wildlife species, anthropogenic disturbances are major determinants of population declines^1^,^2^ and have been related to increased extinction risk^3^. Although natural resource extraction is a major disturbance that can affect wildlife populations before^4^, during^5^, as well as following exploitation activities^6^, the environmental implications specific to broad-scale open-pit (surface) mining are largely unknown.

Open-pit mining has a long history in developed countries around the world^7^,^8^,^9^ and is increasingly expanding in developing countries^10^,^11^. During active open-pit mining, natural vegetation is stripped, the soil layer removed and blasting operations expose target mineral deposits sometimes located hundreds of metres below the original land surface. Mined landscapes are drastically disturbed sites hosting a complex array of features including active and inactive pits, haul roads used by heavy machinery, processing plant(s), offices, and tailing ponds.

Potential ecological impacts of mining include loss of forest cover, habitat fragmentation, changes in topographic complexity and associated alteration of soil, carbon sequestration potential and biodiversity^12^. Although actual disturbance by mining occupies a small geographic range (<1% of the world’s surface), influences of mining can occur at the landscape-level and broad geographic scales^13^. Further, 75% of world mines and mine exploration areas overlap regions with high conservation value and with watershed stress^14^. For example, Borneo forests of global biodiversity significance are under ongoing threat from expansion of open-pit mining^15^.

Most studies that have estimated the environmental effects of mining have done so at the landscape level by quantifying land-cover change^16^, or by assessing effects on aquatic biota^17^. A broad overview on faunal recolonization of mined lands in Australia found that density and richness of mammalian species on reclaimed mines are generally lower than in neighbouring undisturbed areas^18^. While the effects of mining activity on mammalian distribution have been studied for a number of ungulate species^19^,^20^,^21^, major gaps remain in knowledge of impacts of mining on terrestrial ecosystems, especially for large mammals and bats^12^. Even the most basic information is lacking on the effects of mining on omnivores, facultative or obligate carnivores.

We chose a threatened population of grizzly bears (*Ursus arctos*) in Alberta, Canada^22^ to study the effects of open-pit mining on the movement ecology of these animals in a landscape with large-scale coal mining operations. While surface mining for coal is not as widely publicized as the “world-renowned” Alberta oil sands exploitation, coal mining operations are experiencing rapid expansion in response to worldwide demand for hydrocarbons. Globally, coal mining has the highest gross mineral production of mining industries^13^, while the effects of mining on fauna remain mostly unknown.

Since the Foothills Research Institute’s Grizzly Bear Program began bear monitoring on and around mines in Alberta, no grizzly bear mortality has been documented directly because of mining. However, the influences of mining on bear movement have thus far not been quantified. We monitored movements of grizzly bears during active coal mining as well as following mine closure and reclamation to quantify space-use patterns and movement behaviour. Such information can assist land-use planning decisions that promote animal persistence, including designating areas for conservation and ensuring movement connectivity^23^,^24^. Because of the intrinsic spatial nature of GPS, the data can be used to guide on-the-ground activities for conservation, development and restoration planning, while minimizing disturbance to focally monitored wildlife.

We used step length (movement rate) as a surrogate for bear response to perceived risk from humans^25^,^26^. Step length represents the Euclidean distance between two consecutive animal relocations acquired via tracking of movement^27^. In studies of animal movement involving GPS tracking, GPS relocation schedules are pre-programmed by researchers at a regular time interval, for example every 6 hours, which allows calculation of distance moved by each radiocollared animal between two subsequent locations for the standardized time period (every 6 hours in this example). Step length is not assumed to depict the actual movement trajectory of the animal, but it is a replicable sampling unit calculated in a discrete time-frame largely driven by GPS radiocollar battery life limitations. Because step length is a continuous variable that estimates how far an animal moves in a pre-specified period of time, it can be analyzed with conventional statistical models such as generalized linear models^28^, generalized additive models^29^ or analysis of covariance^30^.

At the onset of our assessment of mining effects on grizzly bear movements, we (1) expected no bear use of the mine sites during the active mining phase because of high levels of human activity at the sites, including operation of heavy machinery year-round and on a 24-h basis, pit blasting, presence of an active haul road and coal processing plant, office and service areas. Conversely, based on the ability of bears to colonize other human-disturbed areas such as logging cutblocks^31^, and the possible attraction to ungulates and/or herbaceous forage on reclaimed mines, we (2) expected that bears would move into formerly mined areas and home ranges might include mine sites after mine closure. We (3) anticipated that bear steps starting in active mining areas would be longer than those in undisturbed areas (tree patches) on mines, reflecting a flight response. We also (4) hypothesized that more steps would be confined to mining boundaries after mining than during the active mining phase. (5) Crossing an operational haul road was expected to trigger an increase in step length and change in habitat use through which bears travel, as a result of moving away from vehicular traffic. Following mine closure, reclaimed mine land within the study area had little human access but high bear food availability such as alfalfa (*Medicago sativa*), clover (*Trifolium* spp.), sweet-clover (*Melilotus* spp.), dandelion (*Taraxacum* spp.), elk (*Cervus elaphus*), bighorn sheep (*Ovis canadensis*) and mule deer (*Odocoileus hemionus*)^32^. Because step length is indicative not only of response to risk factors but also feeding, we (6) expected step length after mine reclamation to be shorter if starting on or near mines.

## Results

### Study Area Level

**Figure 1:**
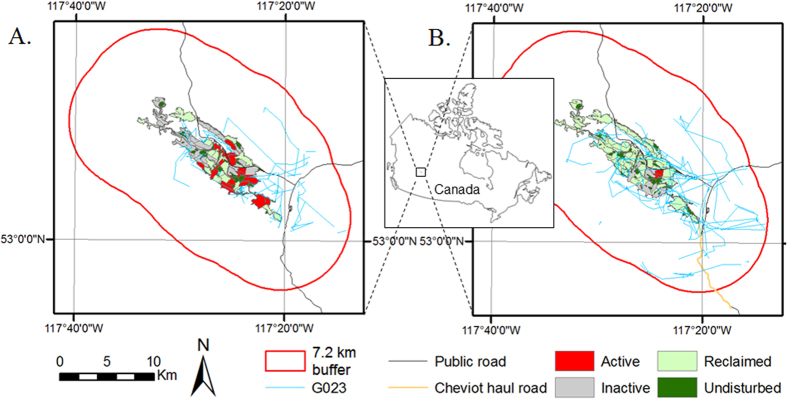
Mineral Disturbance Limits (MDLs) of two neighbouring open-pit coal mines (Luscar and Gregg River) in west-central Alberta, Canada where grizzly bear movement was investigated. MDLs comprise all color-coded polygons depicting mining activity status. A buffer with 7.2 km radius was delineated around MDLs to separate bear movements on and in the vicinity of MDLs from those far from the MDLs (control). The buffer radius represents a distance equal to the 95th percentile of the greatest step length of GPS collared grizzly bears which used the MDLs at least once in the monitoring period. Two insets are provided for the median years of grizzly bear data availability: during (A-2001) versus after (B-2009) mining to illustrate the dynamic nature of mining activity. Four-hour path segments of a female grizzly bear with cubs (G023) are provided for 2001 and 2009 as an example. The map was generated in ArcGIS 10.1 (ESRI, Redlands, USA).

**Figure 2:**
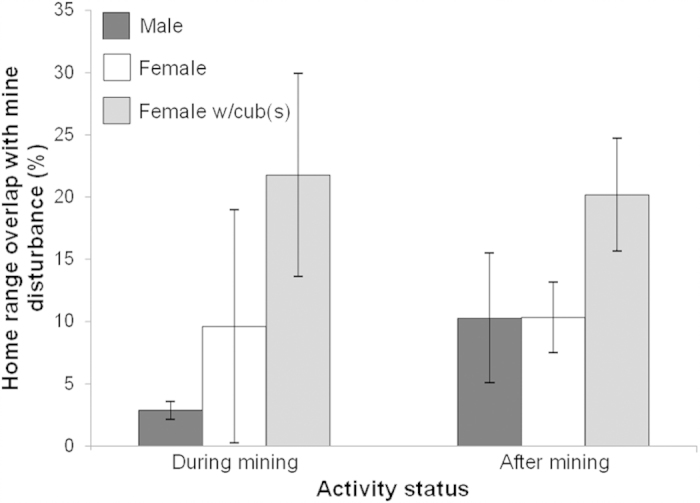
Mean annual home range overlap (95% fixed kernel) with Luscar and Gregg River combined mineral disturbance limits, Alberta, for grizzly bears monitored during (N_*males*_ = 3, N_*females*_ = 3, N_*females with cubs*_ = 3) and after mining (N_*males*_ = 2, N_*females*_ = 4, N_*females with cubs*_ = 3). Sample sizes include only individuals for which home ranges actually overlapped MDLs (overlap >0%). During mining data were collected in 1999–2003, whereas after mining data were collected in 2006 and 2008–2010. Error bars represent ± SD.

**Figure 3:**
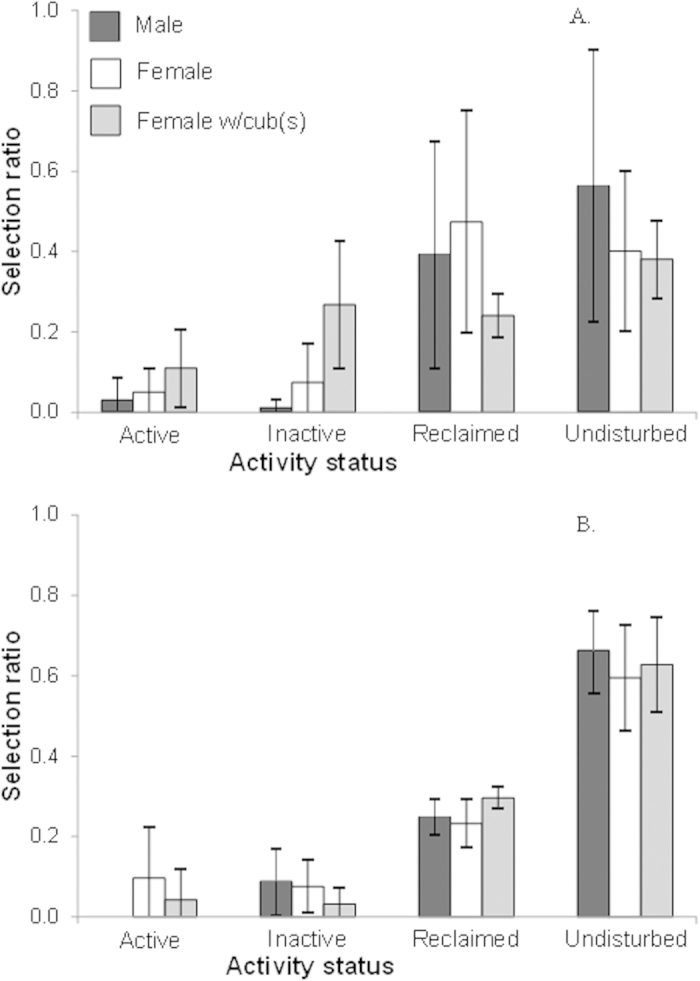
Grizzly bear selection of mining status land categories within the merged mineral disturbance limits of Luscar and Gregg River mines, Alberta. Data were acquired (**A**) during (N_*males*_ = 3, N_*females*_ = 3, N_*females with cubs*_ = 3) and (**B**) after mining (N_*males*_ = 2, N_*females*_ = 4, N_*females with cubs*_ = 3). Sample sizes include only individuals for which home ranges actually overlapped MDLs (overlap >0%). Selection ratios were calculated for each bear and mining status class by dividing the number of used locations by the number of available locations. Error bars represent ± SD.

Grizzly bears of all reproductive classes used the mining area delineated by the Mineral Disturbance Limits (hereafter, MDLs) (Fig. 1) during and after the active mining phase. Mean home range overlap with the MDLs was low for males (mean ± SD, during: 2.9 ± 0.7 km^2^, *n* = 3; after: 10.3 ± 5.2 km^2^, *n* = 2) and single females (during: 9.6 ± 9.3 km^2^, *n* = 3; after: 10.3 ± 2.8 km^2^, *n* = 4), but high for females with cubs (during: 21.8 ± 8.2 km^2^, *n* = 3; after: 20.2 ± 4.5 km^2^, *n* = 3) (Fig. 2). Male home ranges overlapped more after mining than during mining (Fig. 2). Grizzly bears in all reproductive classes selected undisturbed (original tree patches) and reclaimed areas within MDLs both during and after mining (Fig. 3). Bears selected strongly for undisturbed areas and moderately strong for reclaimed areas regardless of reproductive class. Nonetheless, in both mining phases bears generally used areas with active mining operations as well as inactive areas heavily disturbed by mining that provided no vegetative foods.

**Figure 4:**
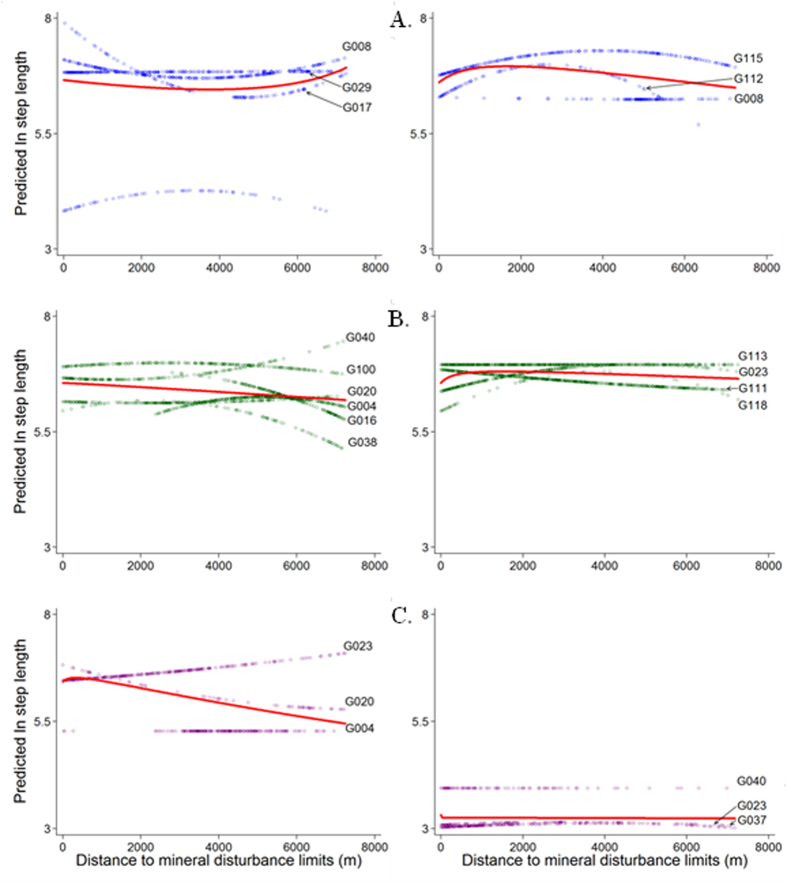
GLM-based predicted values of ln-transformed grizzly bear steps during (left panels) versus after mining (right panels), based on varying distance to mineral disturbance limits from start location of steps. Data include empirical movement steps that occurred within and outside the 7.2 km buffer around the disturbance, during (N_*males*_ = 4, N_*females*_ = 6, N_*females with cubs*_ = 3) and after mining (N_*males*_ = 3, N_*females*_ = 4, N_*females with cubs*_ = 3). Red curves represent reproductive class-level fit for males (**A**), females (**B**), and females with cubs (**C**).

Mine closure caused a significant change in bear movements for males (BACI, *t* = 7.93, *df* = 1383, *P* < 0.0001), females (BACI, *t* = −37.59, *df* = 3232, *P* < 0.0001), and females with cubs (BACI, *t* = 31.93, *df* = 1858, *P* < 0.0001). Contrasting bear movement rates within a 7.2 km buffer from the MDL during versus after mining, while accounting for lengths of steps occurring beyond 7.2 km (control), showed differential response to mining by bear reproductive class. Males had shorter steps within the buffer after mining compared to during mining operations. Conversely, females with/without cubs had longer steps within the buffer after mining compared to during mining. Models incorporating bear steps occurring within the buffer and a suite of predictor variables (supporting Table S2) did not effectively predict reproductive class-level step length during the mining phase (supporting Table S3). However, when step length was analyzed after mining, distance to MDL at the start location of the step was the variable for which the confidence intervals did not overlap zero most consistently, with closer distance corresponding to shorter steps for males and females with cubs, but not for single females (supporting Table S4). Open land cover type at start location corresponded to longer steps for single females, and steps of females with cubs that started close to major roads were more likely to be shorter (supporting Table S4). ‘During’ mining there was substantial variability in predicted step length for individual bears within a given reproductive class depending on distance to MDL, with less variation ‘after’ mining for single females and females with cubs (Fig. 4).

### MDL Level

Some bears were more likely to take longer steps when movement started in an area with active mining operations (supporting Table S5), but this pattern held at the reproductive class-level only for single females in the after mining phase and for the only male included in analysis for the during mining phase (Table 1). Conversely, females with cubs took shorter steps from active mining polygons compared to steps from undisturbed areas during mining. After mining, females with cubs took longer steps from inactive and reclaimed areas than they did from undisturbed areas. Steps from reclaimed polygons also were longer than those from forest areas within the MDL for single females.

**Figure 5:**
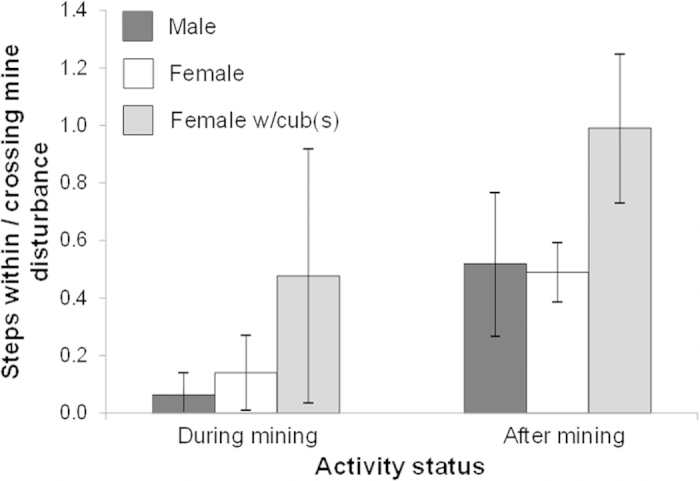
Grizzly bear movements in relation to Luscar and Gregg River merged mineral disturbance limits, given as a ratio of steps within disturbance boundary to steps crossing the disturbance limit but starting and/or ending outside the boundary. Data were available during (N_*males*_ = 4, N_*females*_ = 4, N_*females with cubs*_ = 4) and after mining (N_*males*_ = 3, N_*females*_ = 4, N_*females with cubs*_ = 3). Error bars represent ± SD.

Irrespective of mining phase, bears had a higher proportion of movement steps that crossed the MDL boundaries than steps that stayed within MDL boundaries. The one exception occurred in the after-mining phase for females with cubs, which had an equal proportion of crossing and within-MDL steps (Fig. 5). However, frequency of steps differed by mining phase for males (χ^*2*^ = 21.05, *df* = 1, *P* < 0.0001), females (χ^*2*^ = 13.14, *df* = 1, *P* = 0.0003) and females with cubs (χ^*2*^ = 16.55, *df* = 1, *P* < 0.0001), with a pattern of more movements within MDLs after mining.

### Mining Haul Road

Males (*n* = 3) crossed the location of the haul road before haul road construction but no male crossing by sampled individuals was recorded during the haul road active phase. Single females (*n* = 1) and females with cubs (*n* = 2) crossed the road in the before active haul road phase, with females also crossing during active coal hauling (single females: *n* = 3; females with cubs: *n* = 2). Although slightly higher for the latter, female step length did not differ significantly before (mean ± SE, 1775 ± 366 m) versus during (2376 ± 284 m) haul road activity (two-sample Wilcoxon rank-sum, *z* = −1.408, *P* = 0.159). Length weighted mean distances from haul road during crossings steps were greater for females during the active mining phase (651 ± 102 m) compared to before haul road construction (399 ± 109 m) (two-sample Wilcoxon rank-sum, *z* = −1.716, *P* = 0.086). Length weighted mean distances along steps from forest edge were slightly greater before (120 ± 26 m) versus during (83 ± 8 m) haul road activity but the difference was not statistically significant (two-sample Wilcoxon rank-sum, *z* = 1.210, *P* = 0.226). Female bears did not move in steeper terrain before (0.168 ± 0.01 ruggedness) compared to during (0.170 ± 0.01 ruggedness) haul road activity (two-sample Wilcoxon rank-sum, *z* = −0.616, *P* = 0.538).

## Discussion

Extensive industrial development activities are transforming natural landscapes across all continents except Antarctica, where an Environmental Protocol is in place that bans all mineral resource extraction^33^. To assist impact assessments and implement environmental mitigation strategies for areas targeted for development, we must first document the extent to which industry is impacting not only persistence but also behaviour of wildlife populations of conservation concern. In this context investigating animal movement to understand space use behaviour is a key topic for ecology because of the direct relevance of movement to conservation^34^.

Higher movement rates can lower predation, as exemplified by animal leaving patches when these become risky^35^. However, shorter step length/lower movement rates also can decrease the chance of mortality in risky landscapes^36^,^37^. Such behavioural insights into animal movement often come from studies in controlled conditions on small-bodied species that are easy to monitor. Yet it is often the highly mobile species exhibiting broad and/or rapid movements where baseline space use data are needed to help direct conservation decisions^38^, as is the case for mammal populations on mined landscapes^12^.

Most studies that have documented mammalian response to open-pit mining have focused on mine sites in North America and Australia, likely because of funding availability and stakeholder demand rather than solely reflecting spatial distribution of mining operations (which are becoming widespread globally). Observational studies in North America have documented variable response to mining by ungulate species, including heavy use of mining area^21^, or mine avoidance^20^. Small mammal diversity on reclaimed mines also varies from high^39^ to low^40^. An experimental study^41^ showed that simulated strip mining disturbance affects elk movements, with disturbed calves moving greater distances and using habitat differently than undisturbed ones. Home ranges of vervet monkeys (*Cercopithecus aethiops*) overlapped rehabilitated mine lands in South Africa^42^, whereas Australian mammals were found to readily recolonize rehabilitated gold mines^43^. Also in Australia, bauxite mines were rapidly colonized by most mammalian species present in the area before mining^44^. In Madagascar, lemurs crossed mine concession land either on ground or using bridges that were artificially erected to maintain movement connectivity^45^.

We assessed how the grizzly bear is coping with the dramatic change caused by open-pit coal mining in its distributional range. To our knowledge, this study represents the first detailed assessment of large omnivore/carnivore space use in relation to mining and control areas and at different mining stages. Contrary to expectations, grizzly bear home ranges overlapped mine sites during both mining phases, with the greatest overlap recorded for females with cubs. The latter result, combined with short female with cub movements when starting in an active mining area (during phase), as well as larger proportion of movements within versus simply crossing mines compared to males and single females, suggest habituation to industrial activity and tolerance towards human disturbance. Strategies employed by females with cubs to navigate a risky landscape with pit blasting, shovelling, coal and overburden removal and transport might require short movements, which are associated with ‘hiding’ behaviours and shy personality traits^29^. By taking shorter steps when accompanied by cubs, females potentially minimize human activity-derived risk, because shorter step lengths/lower movement rates decrease predation^36^,^37^. The long steps taken by male bears during active mining are possibly indicative of an alternative strategy to cope with human disturbance, which involves departure from risky patches^35^.

Given that males had greater home range overlap and shorter steps on/near mines following reclamation, whereas female were present on mined areas during and after active mining, it is possible that mining could influence the incidence of encounters between males and females with cubs. Concentrated movements on reclaimed mines for grazing on introduced legumes suggest a risk for females with cubs to encounter possible infanticidal males, which could work against the goal of reclamation to enhance wildlife populations. Although data on outcomes of such encounters is lacking for our study population, infanticide by males can impact cub survival in brown bear populations^46^,^47^. Longer steps of females following reclamation (when a control area was considered) might indicate flight response upon detection of a possible danger such as a male bear, or human trail user^48^. Alternatively, they could indicate movements that are unconstrained by active mining operations, allowing bears of all reproductive classes to effectively graze herbaceous material sown on mines for reclamation. We suggest the need to further study the relationship between males and females with cubs on industrially reclaimed landscapes that change food distribution, at the minimum by monitoring cub survival.

To access reclaimed areas that provided herbaceous forage (legumes and monocots cultivated as part of reclamation), bears needed to cross inhospitable areas dominated by barren rock and/or used by mining activity. In our study system, inactive pit walls on the mine sites are used by bighorn sheep as escape terrain^19^ and some individual bears may be seeking access to this protein-rich resource. Nonetheless, most bears selected undisturbed tree patches when within the MDL, even at the after mining stage when direct human disturbance was minimal. Such patches possibly provide opportunities to hunt ungulates^49^ and/or hiding refuge.

We were unable to ascertain whether the lack of male crossing of the Cheviot haul road during active operation has biological reasons, or is due to sample size limitations regarding number of male bears monitored. In the Yellowhead Ecosystem, which comprises our study area, females cross roads more frequently than males^50^, whereas major highways are crossed by males but not by females^51^. The more directional movement of females when crossing an active haul road, as compared to movement in the absence of the haul road, suggests a slight avoidance of mining traffic.

Females with cubs moved shorter distances when in the vicinity of main public roads than when far from roads, suggesting cautious movements and/or exploiting vegetative foods near roads^52^. These results corroborate the response of females with cubs to roads used by petrocarbon and logging industries^53^ but are opposite the findings of a study in which females were the most risk averse to vehicular traffic^54^.

Female jaguars (*Panthera onca*) used undisturbed areas away from roads whereas males used agricultural lands and roaded areas in addition to undisturbed areas^55^. Similarly, Eurasian lynx (*Lynx lynx*) females with kittens were further from human habitations than males, but this decreased with kitten age^56^. For grizzly/brown bears, results have been mixed with females documented to avoid human activity^54^ or with different sexes responding similarly to human disturbance^48^. Our data are indicative of higher adaptability to mining activity by females with cubs compared to single females or males. However this adaptation may come at the cost of increased risk, given that humans are a major cause of grizzly bear mortality^57^. Minimizing the risk of mortality for bears on active mining landscapes, particularly female bears with cubs using these sites more than other reproductive classes, might potentially be achieved by enforcing speed limits on coal haul roads to decrease the chance of collisions with bears and other wildlife (see Supplementary video), careful waste management and employee and contractor bear safety training. Because habituation to human activity can exacerbate wildlife-human conflicts^58^, we suspect that regulating human access and firearm restrictions on reclaimed mines following mine closure might minimize mortality risk for female grizzly bears with cubs that have adapted to mined lands.

Grizzly bears inhabiting a landscape heavily impacted by large scale industrial development used mines during and after active mining operations. While bears were able to persist on and near mines, their movements were affected by mining. Wilderness areas surrounding mines (including a provincial and national park) were generally used extensively by collared bears, and no bear home range occurred entirely within the perimeter of mineral disturbances. Although bears moved in active and inactive areas, reclaimed and especially undisturbed areas on mine sites were strongly selected, suggesting the importance of maintaining original tree patches in mine planning.

Wildlife recolonization of disturbed lands will likely be most effective if habitats are rapidly restored and human activity is kept to the minimum on these lands following closure of industrial activities. Data on effects of mining across taxa and wildlife recolonization of mine sites are necessary for different study locations as we are only starting to understand the scale of industrial impacts on natural environments. Although based on a 9-year dataset, the results documented in this study are based on relatively small sample sizes (number of individual bears collared) and should thereby be treated with caution. Individual variability in movement was high ‘during’ mining, whereas ‘after’ mining movement patterns of bears were more consistent within reproductive class. This suggests that in periods of active development in particular, individuals might employ different life history strategies to cope with disturbance. Given low densities of grizzly bears in this threatened population (4.79/1,000 km^2^ 59), our data were best available, with a substantial proportion of bears in the region being monitored with GPS telemetry in this study.

Monitoring reproductive parameters and survival would enable a more comprehensive understanding of mining effects on bears and/or other focal species of conservation concern. From an ecosystem-level perspective it would be beneficial to also assess the effects of mining on forest interior habitat specialists such as clouded leopard (*Neofelis nebulosa*), fisher (*Martes pennanti*), arboreal marsupials and many primate species. Similarly, additional understanding is needed for wildlife use and/or recolonization of other drastically modified habitats, such as logging clear cuts or agricultural crops.

Beyond documenting the response of an omnivorous mammal to mining, we hope that this paper and spatially explicit framework herein prove useful in future research on wildlife response to human disturbance. Home range estimation, calculation of habitat selection ratios and basic movement metrics are easily implementable provided GPS animal tracking data and a GIS platform are available. Step length modelling is also achievable in many statistical software packages. Finally, the scaling from individuals to reproductive class (male, female, female with cubs) as performed herein is essential, enabling fine scale understanding of individual behaviour as well as reproductive class-level generalization for conservation.

## Methods

We carried out the study in west-central Alberta, Canada (approximate central coordinates of the study area 53°05′ N 117°25′ W) (Fig. 1). The area has complex topography and land cover, including foothills and the eastern slopes of the Rocky Mountains (elevation; mean ± SD, 1,981 ± 460 m).

Natural vegetation cover is dominated by boreal forest composed of lodgepole pine (*Pinus contorta*), white (*Picea glauca*) and black spruce (*P. mariana*), balsam fir (*Abies balsamea*) and subalpine fir (*A. lasiocarpa*). Mixed and deciduous forest patches dominated by balsam polar (*Populus balsamifera*) and trembling aspen (*P. tremuloides*) are uncommon. Shrub cover is dominated by willow (*Salix* spp.), dwarf birch (*Betula* spp.) and alder (*Alnus* spp.). Grasslands are primarily human-generated and located mostly on open-pit coal mining areas delineated by the MDLs. Within MDLs, disturbance from mining operations occurred 24-h a day during the active mining phase. The extent of grassland varies depending on reclamation stage of the two neighbouring mines under study (Luscar and Gregg River, combined MDL 41.6 km^2^). Barren (non-vegetated) land is present on mines as a result of mining operations and includes pit walls, rock piles/dumps and wide mining roads. Barren land also is naturally present at high elevation where climatic conditions make it difficult for vascular plants to develop.

The study area included the MDLs, adjacent lands up to a distance of 7,240 m from the boundaries of the two neighbouring mines (Fig. 1) as well as a control area beyond this distance. We obtained the spatial extent through buffering the combined MDLs of the two mines by a distance equal to the 95^th^ percentile of the greatest step length of GPS collared grizzly bears which used the MDLs at least once in the monitoring period (*n* = 18) (detail on grizzly bear data in the supporting information). Grizzly bears were captured and handled according to University of Alberta and University of Saskatchewan protocols for Animal Care and Use. All experimental protocols were approved by these two institutions, with protocols reviewed annually by the institutional Animal Care and Use committees. University of Alberta GPS radiocollar fix acquisition schedules varied during the study period and for consistency we rarefied the data to 4-h for the purpose of analyses (details in the supporting information).

An additional coal mine (Cheviot) located 7.9 km away from the other two mines was active during 2004–2010 but we did not consider it for statistical analyses because of small associated bear movement sample sizes. However, we included in analyses the Cheviot mine haul road section located outside MDL boundaries (10.6 km), which links Cheviot active pits to a coal processing plant on Luscar mine.

Public access is prohibited on mine haul roads, which accommodate a range of vehicle sizes including heavy coal haul trucks. County roads (primarily gravel) and jeep trails present in the area have unrestricted use, supporting human recreational activities. Access is not allowed within MDLs except along a few restricted trails. Gas exploration and logging occur to a small extent within the study area outside MDLs.

We used a combination of basic analyses and a two-stage a priori model selection approach to relate covariates to movement for individuals bears and by reproductive class (supporting information). We compared bear movement response to mines by reproductive class and at 3 different spatial levels (study area, within MDLs, and haul road). At the study area level, we estimated bear home range overlap with MDLs, calculated selection ratios for MDLs and modelled step length as a function of environmental covariates (Table S1). At the MDL level, we investigated step length based on mining activity status at the start location of bear steps, as well as compared movements within versus intersecting MDLs. Lastly, we compared bear movements in relation to a major mining haul road before versus during haul road activity (details on all methodological procedures in supporting information). For study area level step length modelling, data were partitioned in two periods using a Before-After-Control-Impact (BACI) design^60^,^61^, with analyses contrasting bear movements during (1999–2003) and after (2006–2010) active mining phases. ‘Impact’ was represented by closure of active mining operations, ‘Before’ was the period of data collection during active mining, the ‘After’ period included years when bears were monitored following closure, and the ‘Control’ consisted of bear movement data acquired in undisturbed areas in the vicinity of mines.

Partitioning was based on Gregg River mine closure in 2004 and completion of extraction operations at the neighbouring Luscar mine after that year. Overburden dumping and sloping/soil placement were still carried out at Luscar mine in small restricted areas, with coal haul occurring along the haul road from Cheviot mine.

## Additional Information

**How to cite this article**: Cristescu, B. *et al.* Large Omnivore Movements in Response to Surface Mining and Mine Reclamation. *Sci. Rep.* **6**, 19177; doi: 10.1038/srep19177 (2016).

## Supplementary Material

Supporting Information

Supplementary Video

## Figures and Tables

**Table 1:** Predicted reproductive class-level grizzly bear step length for bears monitored during (1999–2003) and after mining (2006–2010) at Luscar and Gregg River open-pit coal mines, Alberta, Canada.

Model variable	Reproductive status								
	Male			Female			Female w/cubs		
	*β*_*i*_	90% CI		*β*_*i*_	90% CI		*β*_*i*_	90% CI	
**During mining**									
Intercept	**5.236**	**4.619**	**5.853**	**5.604**	**4.163**	**7.044**	**5.838**	**5.290**	**6.386**
Active	**1.841**	**0.397**	**3.285**	1.109	−0.786	3.004	−**0.298**	−**0.346**	−**0.249**
Inactive	**3.061**	**2.155**	**3.966**	0.357	−0.863	1.576	0.833	−0.115	1.782
Reclaimed	**0.849**	**0.179**	**1.518**	0.699	−0.357	1.754	0.066	−1.153	1.284
**After mining**									
Intercept	**6.064**	**5.554**	**6.573**	**5.643**	**5.119**	**5.807**	**5.493**	**5.310**	**5.676**
Active				**1.320**	**0.473**	**2.167**	−0.843	−2.230	0.544
Inactive	1.911	−0.150	3.971	0.711	−0.044	1.467	**0.544**	**0.050**	**1.038**
Reclaimed	−0.082	−0.655	0.492	**0.740**	**0.308**	**1.171**	**0.528**	**0.444**	**0.612**

Step length predictions are a function of mining status at the start location of steps within MDLs. GLM-based predictions included ‘long’ steps not associated with ungulate consumption or bedding behaviours as identified from GPS cluster investigations (step length by reproductive class: males ≥160 m, females ≥146 m, female with cubs ≥154 m). Significant terms are given in bold. Steps starting in the undisturbed mining category were withheld as base category. No coefficient reporting corresponds to no steps starting in the respective mine activity class.
