# Supplementary material for: Large Omnivore Movements in Response to Surface Mining and Mine Reclamation
Source: Sci Rep. 2016 Jan 11;6:19177. doi: 10.1038/srep19177 (PMC4707505; doi:10.1038/srep19177)
Supplement: Supporting Information [file srep19177-s1.doc]

**Supporting information**

**Large Omnivore Movements in Response to Surface Mining and Mine Reclamation**

Bogdan Cristescu, Gordon B. Stenhouse, Mark S. Boyce

**Methods**

***Grizzly Bear Data***

In 2008-2010, we captured and deployed telemetry-capable GPS collars (ATS, Isanti, USA; Followit, Lindesberg, Sweden) on grizzly bears in the broader Yellowhead Ecosystem region, with support from the Foothills Research Institute Grizzly Bear Program (Hinton, Alberta), who also provided data for 1999-2003 and 2006. Twenty-seven adult individuals (>4 years old, based on cementum annuli from a premolar extracted at capture) were monitored in the area during the study period, of which 18 were included in analyses because of sample size limitations. Analyses included 12 bears monitored during mining (*n_males_* = 5; *n_females_* = 6; *n_females with cubs_* = 5), and 10 bears monitored after mining (*n_males_* = 4; *n_females_* = 4; *n_females with cubs_* = 3). Some females were accompanied by cubs in some years, while being single in other years of a specific mining phase, and were distinctively classified as such for analyses. Monitoring duration was slightly higher during compared to after mining for males (during: mean*_days_* = 135, range = 111−154; after: mean*_days_* = 110, range = 34−119), females (during: mean*_days_* = 167, range = 121−187; after: mean*_days_* = 145, range = 116−176), and females with cubs (during: mean*_days_* = 154, range = 121−201; after: mean*_days_* = 128, range = 103−156). Three individuals (females with/without cubs) were monitored both during and after mining.

To minimize potential bias related to possible differences in bear response to a specific capture technique, we used a variety of capture methods including aerial (helicopter darting) and ground capture (culvert traps and minimal leg-hold snaring)^1^. The Foothills Research Institute Grizzly Bear Program field protocol requires that all collars are removed from the animals at the end of the monitoring period through the use of remote drop-off units or rot-off devices on the collar belting, with animal recapture for collar retrieval being the last option. GPS fix rate varied across collars, with most of the during active mining relocation fixes acquired at 4-h. Therefore, we rarefied all GPS data to this fix interval and connected relocations with straight lines representing movement steps of bears outside winter denning. This resulted in a series of movement segments representing a sample of the movement path for each bear during each year.

***Study Design and GIS Data***

We defined active mining as vegetation clearing, soil disturbance, overburden blasting, overburden/coal removal and overburden dumping. Available annual land cover layers (30-m grain) were mosaicked with corresponding land cover classification that we performed based on detailed knowledge of the area, coupled with overlaying high resolution orthorectified aerial photos and SPOT imagery (Systeme Pour l'Observation de la Terre/French Earth Observation System) available for 2001, 2004, 2007 and 2010. These temporal corrections to land cover were necessary to reflect the dynamic nature of mined landscapes which incorporate cover types that may receive erroneous spectral reflectance scores in regional scale land cover classification. To make habitat categories more relevant to hypothesized perceived security by bears in terms of landscape openness/visibility, seven land cover categories from the original classification were reclassified to two land cover types including closed (forest) and open (shrub, grassland, and barren land). Edge between closed and open land cover classes was extracted from the rasters and used to calculate Euclidean distance to edge. Terrain ruggedness was derived from a DEM (Digital Elevation Model), using a published equation^2^. The equation relates aspect variation and mean slope in a circular 300-m moving window, a size shown to influence grizzly bear occurrence^2-3^. Closed forest^4^, distance to edge^5^ and rugged terrain^6^ were used as surrogates for perceived security by bears. Distance to major public roads and distance to Mineral Disturbance Limit (MDL) also were calculated, with the latter being a key variable in our assessment of mining effects on bear movements. Lastly, polygons depicting mining activity status were updated every year based on consultation with the mining companies and coded as either active, inactive, reclaimed, or undisturbed.

We used STATA v.11.2 (StataCorp, College Station, Texas, USA) for statistical analyses and an alpha level of 0.1 because of expected large environmental variability affecting lengths of movement steps.

***Study Area Level***

*Ranging Patterns and Selection of MDLs*

Using 95% of GPS radiocollar relocations for each bear, we created annual 95% fixed kernel home range polygons^7-8^ using Hawth's Analysis Tools for ArcGIS^9^. The reason for only including 95% of relocations in home range delineation was because our goal was to estimate the area where an animal is expected to be found 95% of the time, thereby excluding extreme areas whose probability of use is expected to be predicted poorly^10^. The home range was estimated by slicing the 95% utilization distribution^11^, which was followed by clipping the estimate by MDL extent and calculating percentage of home range that overlapped the mining area. We then generated random locations at a density of 30 locations/km^2 12^ and identified mining status for both used locations and random locations for the respective year using the Intersect function. We calculated selection ratios for each bear and mining status class by dividing the number of used locations by the number of available locations^13^. To avoid the potential influence of large but seldom used 'habitats', selection ratios were standardized to a sum of 1 by dividing the selection ratio for each mining status class by the sum of selection ratios across all 4 status classes.

*Step Length Modelling*

Rather than relying on a statistical model (such as broken-stick approach) to identify cut-offs in empirical step length distributions presumably indicative of bear behaviour, we used radius data from GPS location clusters with known bear activity to separate short and long bear movement steps by sex and reproductive class. Short steps are likely associated with concentrated bear activity such as bedding or handling an ungulate carcass, differing from long steps that are more indicative of travel or vegetation consumption. The cut-off in step length was calculated based on the maximum GPS cluster diameter as identified from field visitation of clusters representing bedding and kill events, and differed slightly for males (160 m), females (146 m) and females with cubs (154 m). Selection of clusters for visitation and diameter calculations were based on a published algorithm^14^, and modified to incorporate a minimum of 3 GPS locations and 50-m initial seed cluster. Because short steps may be more prone to GPS error^15-16^, and distance to MDL was not a statistically significant covariate for male, female or female with cubs step lengths, we focus hereafter on modelling long movement steps.

The modelling framework followed a two-step approach^17^, which in our case derived reproductive class-level inferences based on individual level models. Only bears with ≥50 4-h movement steps within a 7.24 km buffer from MDLs for any given year were included in statistical analyses. We used generalized linear models (GLMs) with natural log step length as an outcome and a suite of 10 *a priori* selected covariate combinations as predictors, running separate models for each bear of a given reproductive class and mining phase (during versus after). When deemed biologically relevant, squared terms and an interaction were included in candidate models (Table S5) and all testing estimated robust standard errors. Because we were concerned that bears may be taking longer steps in mountainous areas than in the foothills in relation to topography, making use of ridges or valleys, which may have masked the effects of distance to MDL, for each bear we tested for differences in mean lengths of steps taken exclusively in mountains, foothills, and those that included both mountains and foothills. Mountains were defined as elevations >1,700 m^4^, and testing of step length differences was carried out using one-way ANOVAs with log transformed step length as variable of interest. Step lengths for some bears did not differ between mountains, foothills, and mountains and foothills (for steps including both elevation classes), whereas in cases where they did differ, Bonferroni post-hoc analysis showed that steps were actually shortest in the mountains. Given that certain individuals within any reproductive class had steps shorter in the mountains during both phases of mining (during versus after), because we sampled bears that used both mountains and foothills, and because terrain ruggedness was included as a covariate in the candidate model set, we consider any topographical bias minimal.

We ranked models for each individual using AICc and because no model received substantial support (*w*_AICc_ <0.9), we used model averaging to obtain coefficient estimates within each individual^18^. Averaging was carried out only for models that received substantial or some support (∆AICc ≤7)^19^ using the equation


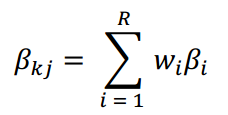


where *β_kj_* is the estimate of coefficient k for individual j, across all i models that received support in model ranking, with *i* = 1,*R*. We then averaged regression coefficients separately across males, females, and females with cubs respectively for during versus after mining, using the following equation^20^


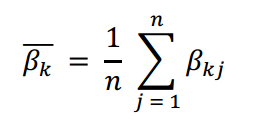
Next, we estimated the variance of each model coefficient using the variation between monitored grizzly bears and the equation


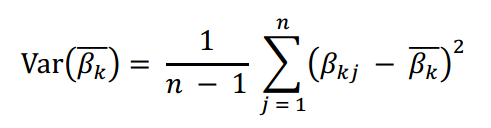
Finally, we computed reproductive class-level 90% confidence intervals separately for each reproductive class and mining phase, based on variance calculated according to equation above.

*BACI Calculations*

A control area where grizzly bear movements were monitored was set beyond a 7.24 km buffer around the MDL. The buffer radius for the control equalled the step length threshold below which most (95%) of the recorded bear step lengths occurred. The threshold was therefore set at the 95^th^ percentile 4-h step length of bears monitored. All bears crossed the 7.24 km buffer perimeter and the control pertained to their movements outside this buffer, being constrained by the finest resolution bear movement data available (4-h). We used standard arithmetic formulas to calculate the normalized (log-transformed) weighted mean difference and standard deviation in step lengths between treatment and control; i.e., between steps potentially influenced by mining (within the buffer) and steps in the control area (outside the buffer). Weights were bear-specific and obtained by dividing the number of steps for an individual by the total number of steps recorded for that specific reproductive class (male, female, or female with cubs). Separate calculations were performed for each reproductive class, mining phase (during, after) and control (control 1: during, control 2: after).

To assess the effect of mine 'manipulation' on bear movements, we used a *t*-test to compare the normalized mean differences in step length during versus after Impact/mine closure. The pooled estimate of the standard deviation (*s_p_*) was


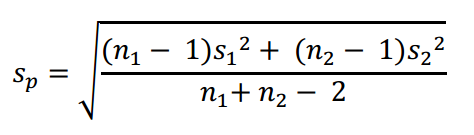

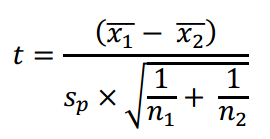
where *n*_1_ is the sample size (number of steps) and *s*_1_ is the standard deviation of step length during mining, *n*_2_ is the sample size and *s*_2_ is the standard deviation after mining. The *t*-statistic to compare the means was

where *x_1_* is the mean difference (treated − control) during mining, and *x_2_* is the mean difference (treated − control) after mining. The calculated *t*-statistic value was compared to the critical value for significance at α = 0.05 with *n_1_*+ *n_2_* - 2 degrees of freedom.

***MDL Level***

*Movements in Relation to Mining Activity Status*

For the extent of mine disturbance limits, we investigated grizzly bear step length as a function of mining activity status (categorical) at the location where a step started. The response variable in generalized linear models with Gaussian family error distribution was log transformed step length data, with separate models created for each individual bear. We restricted the analysis to bears with at least 20 steps starting within the MDL. Regression coefficients were averaged across bears in each reproductive class for during versus after mining, using the same procedure described above under Study Area Level Step Length Modelling. We verified the assumption of normal distribution of deviance residuals by creating histograms and overlaying a normal distribution on the frequency bins. Robust standard errors were used to account for potential misspecification of the family distribution.

*Movements in Relation to MDL Perimeter*

We separated bear steps into two categories: 1. steps that intersected the MDL having start and end locations outside the MDLs, or either the start/end location outside the MDL; and 2. steps that were entirely within mining boundaries. We created an index of movement by taking the ratio of number of steps entirely within the MDL to steps intersecting the MDL. Counts of steps in the two categories were contrasted during and after mining by performing chi-square tests for each bear reproductive class (male, female, female with cubs).

***Mining Haul Road***

We computed all Cheviot mine haul road crossings by intersecting the bear movement paths with a polyline feature representing the haul road location. Haul road construction occurred in 2004 for which no bear movement data were available. Crossings were binned in before (1999-2003) versus during haul road presence and mine traffic (2006 and 2008-2010). To minimize confounding effects related to mining activity within the MDL on bear crossings, analyses were restricted to the haul road section located outside the mine boundaries (10.6 km). We extracted length-weighted mean distances to haul road, forest edge, and terrain ruggedness along steps and compared them before versus during haul road activity using two-sample Wilcoxon rank-sum (Mann-Whitney) tests. Because data on haul road crossing by males during the active mining phase were unavailable, we excluded males from analyses. Female data were pooled across reproductive class categories because of small sample sizes. Length-weighted means were computed by dividing each movement step into segments that passed through single raster cells. The length of each segment was multiplied by the value of the raster cell, summed across the entire step, and divided by the total step length.

**Literature Cited**

1. Cattet, M., Boulanger, J., Stenhouse, G., Powell, R. A. & Reynolds-Hogland, M. L. An evaluation of long-term capture effects in ursids: Implications for wildlife welfare and research. *J. Mammal.* **89**, 973-990 (2008).
2. Nielsen, S. E. et al. Modelling the spatial distribution of human-caused grizzly bear mortalities in the Central Rockies Ecosystem of Canada. *Biol. Conserv.* **120**, 101-113 (2004).
3. Theberge, J. C. Scale-dependent selection of resource characteristics and landscape pattern by female grizzly bears in the eastern slopes of the Canadian Rocky Mountains. Ph.D. dissertation, University of Calgary, Calgary, Canada (2002).
4. Munro, R. H. M., Nielsen, S. E., Price, M. H., Stenhouse, G. B. & Boyce, M. S. Seasonal and diel patterns of grizzly bear diet and activity in west-central Alberta. *J. Mammal.* **87**, 1112-1121 (2006).
5. Ordiz, A., Stoen, O.-G., Delibes, M. & Swenson, J. E. Predators or prey? Spatio-temporal discrimination of human-derived risk by brown bears. *Oecologia* **166**, 59-67 (2011).
6. Nellemann, C. et al. Terrain use by an expanding brown bear population in relation to age, recreational resorts and human settlements. *Biol. Conserv.* **138**, 157-165 (2007).
7. Worton, B. J. Kernel methods for estimating the utilization distribution in home-range studies. *Ecology* **70**, 164-168 (1989).
8. Seaman, D. E. & Powell, R. A. Accuracy of kernel estimators for animal home range analysis. *Ecology* **77**, 2075-2085 (1996).
9. Beyer, H. L. Hawth's Analysis Tools for ArcGIS. [online] URL: <http://www.spatialecology.com/htools> (2004).
10. Powell, R. A. & Mitchell, M. S. What is a home range? *J. Mammal.* **93**, 948-958 (2012).
11. Kie, J. G. The home-range concept: are traditional estimators still relevant with modern telemetry technology? *Phil. Trans. R. Soc. B* **365**, 2221-2231 (2010).
12. Northrup, J. M. et al. Vehicle traffic shapes grizzly bear behaviour on a multiple-use landscape. *J. Appl. Ecol.* **49**, 1159-1167 (2012).
13. Manly, B. F. J., McDonald, L. L., Thomas, D. L., McDonald, T. L. & Erickson, W. P. *Resource selection by animals: statistical design and analysis for field studies.* 2^nd^ ed, Kluwer Academic Publishers, Dodrecht, The Netherlands (2002).
14. Knopff, K. H., Knopff, A. A., Warren, M. B. & Boyce, M. S. Evaluating Global Positioning System telemetry techniques for estimating cougar predation parameters. *J. Wildl. Manage.* **73**, 586-597 (2009).
15. Frair, J. L. et al. Scales of movement by elk (*Cervus elaphus*) in response to heterogeneity in forage resources and predation risk. *Landsc. Ecol.* **20**, 273-287 (2005).
16. Jerde, C. L. & Visscher, D. R. GPS measurement error influences on movement model parameterization. *Ecol. Appl.* **15**, 806-810 (2005).
17. Fieberg, J., Matthiopoulos, J., Hebblewhite, M., Boyce, M.S. & Frair, J. L. Correlation and studies of habitat selection: problem, red herring or opportunity? *Phil. Trans. R. Soc. B* **365**, 2153-2312 (2010).
18. Symonds, M. R. E. & Moussalli, A. A brief guide to model selection, multimodel inference and model averaging in behavioural ecology using Akaike's information criterion. *Behav. Ecol. Sociobiol.* **65**, 13-21 (2011).
19. Grueber, C. E., Nakagawa, S., Lewis, R. J. & Jamieson, I.G. Multimodel inference in ecology and evolution: challenges and solutions. *J. Evol. Biol.* **24**, 699-711 (2010).
20. Marzluff, J. M., Millspaugh, J. J., Hurvitz, P. & Handcock, M. S. Relating resources to a probabilistic measure of space use: Forest fragments and Steller's Jays. *Ecology* **85**, 1411-1427 (2004).

**Table S1.** Remote sensing and GIS covariates used for modelling grizzly bear step length in response to open-pit mining in west-central Alberta, Canada. These covariates were hypothesizes to be linked to perceived security and were tested for grizzly bear steps taken within a 7.24 km buffer from the Mineral Disturbance Limits (MDLs).

| **Model variable** | **Variable code** | **Variable type** | **Unit/Scale** | **Range** |
| --- | --- | --- | --- | --- |
| Land cover |  |  |  |  |
| Closed (forest) | Cov1 | Categorical | n.a. | 0 or 1 |
| Open (shrub, grass, barren) | Cov2 | Categorical | n.a. | 0 or 1 |
| Distance to edge | Dedge | Non-linear | Meter | 0−1,449 |
| Terrain ruggedness index | TRI | Non-linear | Unitless | 0−0.583 |
| Distance to main road | Droad | Non-linear | Meter | 0−11,376 |
| Distance to MDL | Dmdl | Non-linear | Meter | 0−7,261 |
| Open × Distance to MDL | Cov2 × Dmdl | Linear | Meter | 0−7,261 |

**Table S2.** Candidate model set for habitat security features hypothesized to influence grizzly bear step length within a 7.24 km buffer around Luscar and Gregg River mine disturbances, Alberta. Numbers under reproductive class heading represent percentages of the respective model type that were included in model averaging across individual bears.

| **Model** | **Model structure** | ***K_i_*** | **Male** | | **Female** | | **Female w/cubs** | |
| --- | --- | --- | --- | --- | --- | --- | --- | --- |
|  |  |  | During | After | During | After | During | After |
| 1 | TRI + TRI^2^ + Cov2 + Dedge + Dedge^2^ + Dmdl + Dmdl^2^ + Droad + Droad^2^ + Cov2 × Dmdl | 9 | 7.69 | 6.25 | 14.29 | 16.67 | 7.69 | 0 |
| 2 | TRI + TRI^2^ + Cov2 + Dedge + Dedge^2^ + Dmdl + Dmdl^2^ | 8 | 7.69 | 12.5 | 14.29 | 16.67 | 7.69 | 0 |
| 3 | TRI + TRI^2^ + Cov2 + Dedge + Dedge^2^ + Droad + Droad^2^ | 8 | 15.38 | 12.5 | 11.43 | 8.33 | 7.69 | 0 |
| 4 | TRI + TRI^2^ + Cov2 + Dmdl + Dmdl^2^ + Cov2 × Dmdl | 7 | 7.69 | 6.25 | 14.29 | 16.67 | 15.38 | 25 |
| 5 | Cov2 + Dedge + Dedge^2^ + Dmdl + Dmdl^2^ + Cov2 × Dmdl | 7 | 7.69 | 6.25 | 5.71 | 0 | 0 | 0 |
| 6 | Cov2 + Dmdl + Dmdl^2^ + Cov2 × Dmdl | 5 | 23.08 | 18.75 | 11.43 | 16.67 | 7.69 | 0 |
| 7 | Cov2 + Droad + Droad^2^ | 4 | 7.69 | 12.5 | 5.71 | 0 | 15.38 | 0 |
| 8 | Dmdl + Dmdl^2^ | 3 | 15.38 | 12.5 | 14.29 | 25 | 7.69 | 25 |
| 9 | Droad + Droad^2^ | 3 | 7.69 | 6.25 | 5.71 | 0 | 23.08 | 50 |
| 10 | Cov2 | 2 | 0 | 6.25 | 2.86 | 0 | 7.69 | 0 |

**Table S3.** GLM-based predicted reproductive class-level step length of grizzly bears as a function of habitat security features (during mining versus control). Movement data were collected during 1999-2003 in west-central Alberta. 'During mining' steps occurred within a 7.24 km buffer from mine disturbance limits, whereas 'Control' steps were recorded beyond 7.24 km. Parameter estimates correspond to 'long' steps not associated with confined bear activity such as ungulate consumption or bedding. Terms for which confidence intervals do not overlap zero are given in bold. Closed (forest) land cover was withheld as reference category.

| **Model variable** | **Reproductive status** | | | | | | | | | | |
| --- | --- | --- | --- | --- | --- | --- | --- | --- | --- | --- | --- |
|  | Male | | |  | Female | | |  | Female w/ cubs | | |
|  | *β_i_* | 90% CI | |  | *β_i_* | 90% CI | |  | *β_i_* | 90% CI | |
| **During mining** |  |  |  |  |  |  |  |  |  |  |  |
| Intercept | **6.513** | **4.928** | **8.097** |  | **6.194** | **5.799** | **6.589** |  | **6.184** | **5.344** | **7.024** |
| TRI | −1.568 | −4.082 | 0.946 |  | 0.616 | −3.121 | 4.354 |  | 3.039 | −1.926 | 8.004 |
| TRI^2^ | 1.562 | −0.770 | 3.894 |  | −6.467 | −17.991 | 5.056 |  | −17.583 | −46.149 | 10.983 |
| Cov2 | 0.111 | −0.050 | 0.272 |  | 0.060 | −0.078 | 0.199 |  | −0.097 | −0.296 | 0.102 |
| Dedge | −0.182^ | −0.369 | 0.004 |  | 0.223^ | −0.147 | 0.592 |  | 1.119^ | −0.725 | 2.963 |
| Dedge^2^ | 0.215^^ | −0.035 | 0.465 |  | −0.037^^ | −0.683 | 0.609 |  | −2.919^^ | −7.730 | 1.893 |
| Dmdl | −0.156^ | −0.488 | 0.177 |  | 0.139^ | −0.009 | 0.287 |  | −0.084^ | −0.272 | 0.104 |
| Dmdl^2^ | 0.017^^ | −0.023 | 0.057 |  | −0.018^^ | −0.038 | 0.002 |  | 0.007^^ | −0.002 | 0.002 |
| Droad | 0.062^ | −0.045 | 0.170 |  | 0.029^ | −0.022 | 0.080 |  | 0.007^ | −0.043 | 0.057 |
| Droad^2^ | −0.005^^ | −0.015 | 0.004 |  | −0.002^^ | −0.005 | 0.002 |  | 0.003^^ | −0.005 | 0.011 |
| Cov2 × Dmdl | −0.019^ | −0.089 | 0.051 |  | −0.006^ | −0.044 | 0.032 |  | 0.027^ | −0.011 | 0.065 |
| **Control** |  |  |  |  |  |  |  |  |  |  |  |
| Intercept | **5.793** | **5.173** | **6.413** |  | **6.870** | **4.989** | **8.750** |  | **6.400** | **6.260** | **6.540** |
| TRI | **4.541** | **0.223** | **8.860** |  | 0.988 | −0.533 | 2.509 |  | −3.633 | −9.609 | 2.343 |
| TRI^2^ | −18.394 | −37.348 | 0.560 |  | −5.195 | −13.427 | 3.038 |  | 19.651 | −12.675 | 51.976 |
| Cov2 | 0.229 | −0.121 | 0.579 |  | −0.011 | −1.649 | 1.628 |  | −0.085 | −0.236 | 0.066 |
| Dedge | −0.024^ | −0.249 | 0.201 |  | **−0.995^** | **−1.657** | **−0.332** |  | 1.497^ | −0.965 | 3.959 |
| Dedge^2^ | **0.278^^** | **0.391** | **0.947** |  | **1.843^^** | **0.008** | **3.678** |  | −4.329^^ | −11.451 | 2.792 |
| Dmdl | 0.035^ | −0.022 | 0.092 |  | −0.302^ | −0.758 | 0.153 |  | 0.055^ | −0.036 | 0.146 |
| Dmdl^2^ | −0.000^^ | −0.000 | 0.000 |  | 0.019^^ | −0.005 | 0.043 |  | −0.003^^ | −0.008 | 0.002 |
| Droad | **0.094^** | **0.015** | **0.173** |  | 0.005^ | −0.039 | 0.049 |  | 0.062^ | −0.006 | 0.130 |
| Droad^2^ | **−0.006^^** | **0.012** | **0.001** |  | −0.001^^ | −0.005 | 0.003 |  | −0.004^^ | −0.014 | 0.005 |
| Cov2 × Dmdl | −0.000^ | −0.002 | 0.002 |  | −0.004^ | −0.193 | 0.185 |  | 0.011^ | −0.007 | 0.028 |

^ Coefficient reported at 10^3^ times its actual value; ^^ Coefficient reported at 10^6^ times its actual value

**Table S4.** GLM-based predicted reproductive class-level step length of grizzly bears as a function of habitat security features (after mining versus control). Movement data were collected during 2006 and 2008-2010 in west-central Alberta. 'After mining' steps occurred within a 7.24 km buffer from mine disturbance limits, whereas 'Control' steps were recorded beyond 7.24 km. Parameter estimates correspond to 'long' steps not associated with confined bear activity such as ungulate consumption or bedding. Terms for which confidence intervals do not overlap zero are given in bold. Closed (forest) land cover was withheld as reference category.

| **Model variable** | **Reproductive status** | | | | | | | | | | |
| --- | --- | --- | --- | --- | --- | --- | --- | --- | --- | --- | --- |
|  | Male | | |  | Female | | |  | Female w/ cubs | | |
|  | *β_i_* | 90% CI | |  | *β_i_* | 90% CI | |  | *β_i_* | 90% CI | |
| **After mining** |  |  |  |  |  |  |  |  |  |  |  |
| Intercept | **6.971** | **6.331** | **7.610** |  | **6.833** | **6.079** | **7.587** |  | **3.522** | **3.082** | **3.962** |
| TRI | 0.150 | −0.044 | 0.344 |  | −2.823 | −8.223 | 2.578 |  | −1.672 | −4.421 | 1.078 |
| TRI^2^ | −2.708 | −6.186 | 0.770 |  | 2.118 | −13.667 | 17.903 |  | 3.360 | −2.167 | 8.886 |
| Cov2 | −0.184 | −0.374 | 0.011 |  | **0.028** | **0.002** | **0.053** |  | 0.004 | −0.003 | 0.011 |
| Dedge | 0.038^ | −0.033 | 0.109 |  | 0.231^ | −0.219 | 0.681 |  | 0 | 0 | 0 |
| Dedge^2^ | −0.194^^ | −0.498 | 0.110 |  | −0.278^^ | −0.934 | 0.378 |  | 0 | 0 | 0 |
| Dmdl | **0.261^** | **0.002** | **0.520** |  | 0.148^ | −0.048 | 0.344 |  | **0.027^** | **0.004** | **0.050** |
| Dmdl^2^ | −0.043^^ | −0.091 | 0.005 |  | −0.019^^ | −0.042 | 0.003 |  | **−0.005^^** | **−0.008** | **−0.001** |
| Droad | −0.084^ | −0.209 | 0.042 |  | 0.043^ | −0.027 | 0.113 |  | **0.020^** | **0.002** | **0.037** |
| Droad^2^ | 0.004^^ | −0.002 | 0.011 |  | −0.002^^ | −0.004 | 0.001 |  | −0.006 | −0.016 | 0.003 |
| Cov2 × Dmdl | −0.005^ | −0.016 | 0.006 |  | −0.001^ | −0.004 | 0.002 |  | 0.008 | −0.005 | 0.021 |
| **Control** |  |  |  |  |  |  |  |  |  |  |  |
| Intercept | **3.871** | **3.137** | **4.604** |  | 6.125 | NA^+^ | NA^+^ |  | NA^++^ | NA^++^ | NA^++^ |
| TRI | −0.090 | −0.239 | 0.058 |  | 0 | NA^+^ | NA^+^ |  | NA^++^ | NA^++^ | NA^++^ |
| TRI^2^ | 0.182 | −0.117 | 0.481 |  | 0 | NA^+^ | NA^+^ |  | NA^++^ | NA^++^ | NA^++^ |
| Cov2 | −0.097 | −0.256 | 0.062 |  | 0.137 | NA^+^ | NA^+^ |  | NA^++^ | NA^++^ | NA^++^ |
| Dedge | 0.022^ | −0.014 | 0.058 |  | 0 | NA^+^ | NA^+^ |  | NA^++^ | NA^++^ | NA^++^ |
| Dedge^2^ | −0.008^^ | −0.021 | 0.005 |  | 0 | NA^+^ | NA^+^ |  | NA^++^ | NA^++^ | NA^++^ |
| Dmdl | **0.222^** | **0.080** | **0.364** |  | 0 | NA^+^ | NA^+^ |  | NA^++^ | NA^++^ | NA^++^ |
| Dmdl^2^ | **−0.005^^** | **−0.009** | **−0.002** |  | 0 | NA^+^ | NA^+^ |  | NA^++^ | NA^++^ | NA^++^ |
| Droad | 0 | 0 | 0 |  | 0.272^ | NA^+^ | NA^+^ |  | NA^++^ | NA^++^ | NA^++^ |
| Droad^2^ | 0 | 0 | 0 |  | −0.014^^ | NA^+^ | NA^+^ |  | NA^++^ | NA^++^ | NA^++^ |
| Cov2 × Dmdl | 0.001^ | −0.001 | 0.003 |  | 0 | NA^+^ | NA^+^ |  | NA^++^ | NA^++^ | NA^++^ |

^ Coefficient reported at 10^3^ times its actual value; ^^ Coefficient reported at 10^6^ times its actual value

^+^ Based on 1 individual animal only; ^++^ Models did not receive support (∆AICc >7)

**Table S5.** Predicted individual-level grizzly bear step length as a function of mining status at Luscar and Gregg River open-pit coal mines, Alberta, Canada (1999-2003, 2006; 2008-2010 data). GLM-based predictions included 'long' steps not associated with ungulate consumption or bedding behaviours as identified from GPS cluster investigations (step length by reproductive class [RC]: males ≥160 m, females ≥146 m, female with cubs ≥154 m). Significant terms are given in bold. Steps starting in the undisturbed mining category were withheld as base category. No reporting of coefficients corresponds to no steps starting in the respective mine activity class.

| **Bear ID** | | **Sex** | | **RC** | | **Intercept** | | | | | **Active** | | | | | **Inactive** | | | | | **Reclaimed** | | | | |
| --- | --- | --- | --- | --- | --- | --- | --- | --- | --- | --- | --- | --- | --- | --- | --- | --- | --- | --- | --- | --- | --- | --- | --- | --- | --- |
|  |  | |  | | *β_i_* | | 90% CI | | | *β_i_* | | 90% CI | | | *β_i_* | | 90% CI | | | *β_i_* | | 90% CI | | |  |
|  | |  | |  | |  | | Lower Upper | | |  | | Lower Upper | | |  | | Lower Upper | | |  | | Lower Upper | | |
| **During mining** | | | | | |  | |  |  | |  | |  |  | |  | |  |  | |  | |  |  | |
| G020 | | F | | Single | | **4.728** | | **3.524** | **5.932** | | **2.261** | | **0.741** | **3.781** | | 1.098 | | −0.214 | 2.410 | | **1.340** | | **0.081** | **2.598** | |
| G040 | | F | | Single | | **6.479** | | **5.817** | **7.140** | | −0.043 | | −1.306 | 1.220 | | −0.385 | | −1.215 | 0.445 | | 0.057 | | −0.894 | 1.009 | |
| G023 | | F | | COY | | **6.171** | | **5.675** | **6.666** | | −0.327 | | −0.974 | 0.321 | | 0.257 | | −0.344 | 0.857 | | −0.675 | | −1.451 | 0.101 | |
| G040 | | F | | COY | | **5.505** | | **4.992** | **6.017** | | −0.268 | | −1.077 | 0.540 | | **1.410** | | **0.759** | **2.062** | | **0.806** | | **0.192** | **1.421** | |
| G029 | | M | |  | | **5.236** | | **4.619** | **5.853** | | **1.841** | | **0.397** | **3.285** | | **3.061** | | **2.155** | **3.966** | | **0.849** | | **0.179** | **1.518** | |
| **After mining** | | | | | |  | |  |  | |  | |  |  | |  | |  |  | |  | |  |  | |
| G023 | | F | | Single | | **5.149** | | **4.597** | **5.700** | | 1.005 | | −0.631 | 2.641 | | **1.951** | | **1.128** | **2.774** | | 0.559 | | −0.045 | 1.162 | |
| G111 | | F | | Single | | **5.778** | | **5.118** | **6.438** | | **2.086** | | **1.426** | **2.746** | | 0.864 | | −0.501 | 2.229 | | 0.533 | | −0.193 | 1.259 | |
| G113 | | F | | Single | | **5.059** | | **4.542** | **5.576** | | **2.188** | | **1.569** | **2.807** | | 0.030 | | −1.341 | 1.402 | | **1.515** | | **0.91** | **2.119** | |
| G118 | | F | | Single | | **5.867** | | **5.259** | **6.475** | |  | |  |  | |  | |  |  | | 0.351 | | −0.303 | 1.005 | |
| G023 | | F | | Yearling | | **5.649** | | **5.247** | **6.052** | |  | |  |  | | **0.597** | | **0.194** | **0.999** | | 0.449 | | −0.017 | 0.915 | |
| G040 | | F | | Yearling | | **5.553** | | **5.072** | **6.034** | | **−2.529** | | **−4.495** | **−0.563** | | **1.036** | | **0.080** | **1.991** | | **0.623** | | **0.071** | **1.174** | |
| G037 | | F | | 2-Yr old | | **5.278** | | **4.942** | **5.613** | |  | |  |  | |  | |  |  | | **0.512** | | **0.135** | **0.889** | |
| G112 | | M | | Male | | **6.373** | | **5.938** | **6.808** | |  | |  |  | | 0.658 | | −0.030 | 1.319 | | −0.43 | | −1.004 | 0.144 | |
| G115 | | M | | Male | | **5.754** | | **5.167** | **6.340** | |  | |  |  | | **3.163** | | **2.577** | **3.749** | | 0.267 | | −0.366 | 0.899 | |

**Video caption.** Female grizzly bear attempting to cross an active coal haul road (Cheviot road, Alberta, Canada). We captured this bear (G118) a few days later and deployed a GPS collar on it to track its movements on and around the mined landscape.

.
